# Type 2 Diabetes Self-Management Behaviors and Glycemic Control Under China’s Diabetes Prevention and Control Action Program

**DOI:** 10.3389/ijph.2025.1608067

**Published:** 2025-05-30

**Authors:** Shuyan Gu, Xiaoyong Wang, Fangfang Shen, Hai Gu, Ning Zhang, Yuan Zhou, Xiaoling Wang

**Affiliations:** ^1^ Center for Health Policy and Management Studies, School of Government, Nanjing University, Nanjing, China; ^2^ Health Insurance Office, Shandong Provincial Hospital Affiliated to Shandong First Medical University, Jinan, China; ^3^ General Practice Department, Daguang Road Community Healthcare Center, Nanjing, China; ^4^ Department of Endocrinology, Nanjing Drum Tower Hospital, Affiliated to Nanjing University Medical School, Nanjing, China; ^5^ Department of Endocrinology, The First Affiliated Hospital of Nanjing Medical University, Nanjing, China; ^6^ Department of Endocrinology, Xinhua Hospital Affiliated to Shanghai Jiao Tong University School of Medicine, Shanghai, China

**Keywords:** diabetes self-management behaviors, glycemic control, type 2 diabetes, health promotion, diabetes prevention and control action

## Abstract

**Objectives:**

To investigate type 2 diabetes self-management behaviors and glycemic control under the impacts of COVID-19 legacy and Diabetes Prevention and Control Action, and explore the heterogeneous impacts of five self-management activities on glycemic control and how these impacts differ across key groups.

**Methods:**

A cross-sectional survey was conducted between April and September 2023 in hospitals and communities in China. Overall, 1817 adults with type 2 diabetes and normal cognitive and behavioral capacities completed a questionnaire regarding diabetes self-management behaviors and glycemic control. Ordinary least squares regression analyses were conducted.

**Results:**

Mean score of overall self-management behaviors was 5.89. About 26.86% reported good glycemic control. Among five self-management activities, medication adherence was the best (mean = 6.77) but glucose-monitoring adherence was the worst (mean = 5.18). Overall self-management behaviors and the five activities (coefficient = 0.031–0.146, all p < 0.001) all exerted positive impacts on glycemic control, with dietary control showing the greatest impact while medication adherence the least. Younger persons, rural persons, and persons with financial difficulties were key groups benefiting less from self-management.

**Conclusion:**

Diabetes self-management behaviors and glycemic control were suboptimal. Customized health promotions should focus on key groups and addressing the deficiencies in self-management activities especially dietary control.

## Introduction

Diabetes confers substantial disease burden worldwide. China has the world’s largest diabetes epidemic with 116 million adults living with diabetes and medical expenditure of USD 109 billion in 2019 [1]. The key to reducing the disease burden is to improve diabetes management. Type 2 diabetes as the most common type of diabetes is a lifelong disease [1]. About 95% of its health management activities belong to the diabetes self-management behaviors of persons with type 2 diabetes [2], which are defined as daily self-care activities taken by them, including glucose monitoring, dietary control, physical activity, and medication adherence [3]. Optimal and persistent diabetes self-management behaviors can improve glycemic control and prevent diabetes complications, thus reducing the disease burden [4].

However, evidence before 2019 showed that diabetes self-management behaviors and glycemic control of persons with type 2 diabetes were suboptimal in China [5–11], while studies also suggested that diabetes self-management behaviors had a positive impact on glycemic control [12, 13]. Thus, China launched the national Diabetes Prevention and Control Action (DPCA) in July 2019, as a key part of the Healthy China Initiative (2019–2030), to promote standardized diabetes management and public health [14, 15]. One of the basic principles and key contents of DPCA is that “standardizing health management is the priority” and the corresponding goals are to raise the standardized management rate of persons with diabetes to at least 70% and to continuously increase the glycemic control rate by 2030 [14, 15]. However, the outbreak of coronavirus disease 2019 (COVID-19) in January 2020 caused upheavals in healthcare services, lockdown measures (e.g., quarantine measures, social distancing, close-contact identification, risk area designation), and health issues, which have largely disrupted diabetes self-management behaviors and glycemic control of persons with type 2 diabetes [16–19]. Currently, the economy, society, and population life are recovering since China dropped COVID-19 lockdown in January 2023 [20, 21], but the impacts of the COVID-19 legacy and the threats of infectious diseases exist in the long-term [22]. Besides, since the national DPCA has been implemented for several years, it is essential to conduct phased evaluations to check its effects and delve into the existing problems, thus providing empirical evidence for optimizing it.

Therefore, in this special phase, investigating the practices of diabetes self-management behaviors and glycemic control of persons with type 2 diabetes and their associations across different population groups is of significance, which can offer valuable insights into how diabetes self-management behaviors and glycemic control have evolved under the combined impacts of the COVID-19 legacy and the national DPCA, while also identifying real-world challenges and key groups in promoting diabetes self-management. However, there is a lack of such evidence in China. Therefore, this study aimed to investigate diabetes self-management behaviors and glycemic control of persons with type 2 diabetes under the combined impacts of the COVID-19 legacy and the national DPCA, and explore the heterogeneous impacts of five self-management activities on glycemic control and how these impacts differ across key groups. By doing so, we provided advice for healthcare providers in tailoring diabetes health promotion interventions.

## Methods

### Design and Sampling

This was a cross-sectional questionnaire survey conducted in persons with type 2 diabetes from April to September 2023 in China. Inclusion criteria were persons aged ≥18 years, with a physician diagnosis of type 2 diabetes, and having normal cognitive and behavioral capacities. Exclusion criteria were persons having severe mental health disorders, having serious illnesses (e.g., liver insufficiency, respiratory failure, or cancer), or in pregnancy. G*Power version 3.1.9.7 was used to calculate the sample size. It indicated that a minimum sample size of 213 was required for linear multiple regression to achieve a power of 0.95, with an effect size of 0.15, an α level of 0.05, and 18 predictors. Considering a potential 20% dropout rate, the required sample size was at least 267. All procedures performed in this study involving human participants were in accordance with the ethical standards of the Medical Ethics Committee of Nanjing University (NO: OAP20230407002) and with the 1964 Helsinki declaration and its later amendments or comparable ethical standards.

This survey utilized a convenience sampling strategy. Participants were recruited through two ways: first, from tertiary hospitals and community hospitals in Nanjing, Shanghai, and Jinan by trained nurses and university students, considering both geographic representation and accessibility for research collaboration; second, from residential communities nationwide by trained university students, based on a university social practice project. The survey was voluntary, anonymous, and confidential, administered through face-to-face questionnaires. Before data collection, permissions were obtained from the hospitals and communities. Investigators, accompanied by institutional staff, approached potential participants, explained the survey aims and procedures, and distributed informed consent forms. After obtaining consent, participants were given the questionnaire and asked to complete it independently and return it in approximately 30 min. Investigators were available to address any questions. For participants who were illiterate or visually impaired, investigators provided neutral help under their consents by reading each item out and recording their verbal answers on the questionnaire. Upon completion of each questionnaire, the investigator collected it and carefully checked it for missing items, which were addressed with the participant in person. Initially, 1830 participants were recruited, but 13 withdrew early and did not return their questionnaires. Ultimately, 1817 participants completed and returned their questionnaires without missing data and were included in this study. The response rate was 99.29%.

### Variables and Measures

The survey was conducted using a self-reported questionnaire developed based on literature review, expert consultation, and a pilot study. Three categories of variables were included.

#### Independent Variables

Diabetes self-management behaviors were measured by the Chinese version of Diabetes self-management Questionnaire (DSMQ), which was originally developed and validated by Schmitt et al with a Cronbach’s α of 0.840 for the original scale [23] and later translated and validated by Li et al in the Chinese cultural context with a Cronbach’s α of 0.764 for the Chinese version [24]. It is a 16-item scale to measure diabetes self-management behaviors in five subscales including dietary control, physical activity, glucose monitoring, medication adherence, and physician contact, along with the sum scale measuring overall self-management behaviors [24]. Participants were asked to self-rate the extent to which each item applied to them over last 8 weeks on a four-point Likert scale. Seven of the items were positively framed regarding what was effective self-management behaviors and scored from 0 (does not apply to me) to 3 (applies to me very much). Nine were negatively framed and scored inversely. The scores of sum scale and subscales were calculated as sums of item scores and then transformed to scores ranging from 0 to 10 (i.e., raw score/theoretical maximum score * 10), with higher scores indicating better self-management behaviors. A cut-off score of ≤6.0 for the sum scale was recommended as indicative of suboptimal self-management behaviors [25, 26]. In this study, the Cronbach’s α for the scale was 0.818.

#### Dependent Variable

Glycemic control was measured by the item: “How is your glycemic control based on your most recent glycated hemoglobin A1c (HbA1c) result, with HbA1c <7.0% defined as good glycemic control?.” Responses were coded from 1 (poor) to 3 (good), with higher scores indicating better glycemic control. A score of 3 was considered to represent good glycemic control.

#### Control Variables

We controlled for participant characteristics based on previous studies [5, 7, 27–30], including age (years), gender (male, female), marital status (having no partner, having a partner), education level (illiterate, primary school, middle school, high school, university or above), medical insurance (no, yes), employment status (retired, unemployed, employed/students), region of residence (rural, urban), living arrangement (living alone, living with others), financial difficulties (no, yes), diabetes duration (years), diabetes family history (no, yes), diabetes complication (no, yes), hypertension (no, yes), dyslipidemia (no, yes), overweight/obesity (no, yes), antidiabetic medication (none, oral medication only, involve injectable medication). Overweight referred to 24 ≤ body mass index (BMI) < 28 kg/m^2^ and obesity referred to a BMI ≥28 kg/m^2^ based on weight criteria for adults in China [31].

### Statistical Analysis

A descriptive analysis was used to describe participant characteristics. Continuous variables were described using means and standard deviations (SD). Categorical variables were described using numbers and percentages. In comparing the differences in diabetes self-management behaviors based on participant characteristics, a t-test or one-way ANOVA was used for categorical variables, and Pearson correlation test was used for continuous variables. Ordinary least squares regression model was used to assess the impacts of diabetes self-management behaviors on glycemic control and the heterogeneity. To test the results’ robustness, the methods of removing the control variable of diabetes duration and converting overall self-management behaviors to a categorical variable (poor, good) based on the cut-off score of ≤6.0 were used [25, 26]. Data were analyzed with Stata SE 16.0 software (Stata Corp, College Station, Texas, United States). A *p*-value of <0.05 was considered statistically significant.

## Results

### Participant Characteristics

A total of 1817 participants with type 2 diabetes were included. The average age was 56.52 (SD = 15.55) years, with a female proportion of 41.66%. Most participants had a partner (85.58%), were educated (95.87%), had medical insurance (97.36%), lived in urban areas (77.88%), and reported no financial difficulties (77.16%). The average diabetes duration was 10.05 (SD = 9.19) years, with 45.02% having a diabetes family history. Among the participants, 77.05% reported having diabetes complications, 58.61% had hypertension, 56.91% had dyslipidemia, and 49.04% were overweight or obese (Table 1).

**TABLE 1:** Participant characteristics and diabetes self-management behaviors according to participant characteristics (China. 2023).

Variables	Participant characteristics	Diabetes self-management behaviors											
		Overall self-management behaviors		Dietary control		Physical activity		Glucose monitoring		Medication adherence		Physician contact	
	n (%)	Mean (SD)	p	Mean (SD)	p	Mean (SD)	p	Mean (SD)	p	Mean (SD)	p	Mean (SD)	p
Age^a^, year	56.52 (15.55)	5.89 (1.70)	<0.001	6.26 (2.41)	<0.001	5.84 (2.50)	<0.001	5.18 (2.50)	0.004	6.77 (2.65)	<0.001	5.82 (1.61)	<0.001
Age group			<0.001		<0.001		<0.001		<0.001		<0.001		<0.001
Younger ones (<60 years)	887 (48.82)	5.44 (1.74)		5.69 (2.41)		5.38 (2.51)		4.94 (2.59)		6.02 (2.73)		5.55 (1.62)	
Older ones (≥60 years)	930 (51.18)	6.33 (1.54)		6.80 (2.27)		6.29 (2.41)		5.41 (2.39)		7.49 (2.35)		6.09 (1.54)	
Gender			<0.001		<0.001		0.279		0.031		<0.001		0.006
Male	1,060 (58.34)	5.74 (1.71)		6.01 (2.40)		5.79 (2.48)		5.07 (2.48)		6.55 (2.68)		5.74 (1.62)	
Female	757 (41.66)	6.11 (1.67)		6.61 (2.37)		5.92 (2.52)		5.33 (2.52)		7.08 (2.57)		5.95 (1.57)	
Marital status			0.071		0.058		0.018		0.795		0.077		0.434
Having no partner	262 (14.42)	5.70 (1.86)		5.98 (2.62)		5.50 (2.61)		5.14 (2.55)		6.48 (2.91)		5.75 (1.77)	
Having a partner	1,555 (85.58)	5.93 (1.67)		6.30 (2.36)		5.90 (2.47)		5.19 (2.49)		6.82 (2.60)		5.84 (1.58)	
Education level			<0.001		0.004		0.038		<0.001		<0.001		0.008
Illiterate	75 (4.13)	5.38 (1.46)		5.91 (2.30)		5.36 (2.39)		4.12 (2.39)		6.11 (2.68)		5.88 (1.38)	
Primary school	199 (10.95)	5.60 (1.55)		6.00 (2.35)		5.46 (2.55)		4.76 (2.32)		6.52 (2.44)		5.67 (1.36)	
Middle school	458 (25.21)	5.97 (1.66)		6.35 (2.47)		5.90 (2.47)		5.17 (2.52)		7.10 (2.52)		5.83 (1.53)	
High school	582 (32.03)	6.10 (1.68)		6.51 (2.34)		6.01 (2.47)		5.33 (2.42)		7.01 (2.63)		6.02 (1.61)	
University or above	503 (27.68)	5.78 (1.81)		6.03 (2.42)		5.83 (2.54)		5.34 (2.61)		6.39 (2.79)		5.65 (1.76)	
Medical insurance			0.025		0.164		0.112		0.198		<0.001		0.330
No	48 (2.64)	5.35 (1.72)		5.78 (2.27)		5.28 (2.45)		4.72 (2.57)		5.38 (3.10)		5.60 (1.89)	
Yes	1769 (97.36)	5.91 (1.70)		6.27 (2.41)		5.86 (2.50)		5.19 (2.50)		6.81 (2.62)		5.83 (1.60)	
Employment status			<0.001		<0.001		<0.001		<0.001		<0.001		<0.001
Retired	955 (52.56)	6.34 (1.54)		6.82 (2.28)		6.34 (2.41)		5.43 (2.36)		7.43 (2.40)		6.10 (1.56)	
Unemployed	156 (8.59)	5.24 (1.62)		5.54 (2.23)		4.81 (2.48)		4.79 (2.48)		6.04 (2.56)		5.36 (1.51)	
Employed/students	706 (38.86)	5.44 (1.76)		5.66 (2.43)		5.40 (2.46)		4.93 (2.66)		6.05 (2.76)		5.55 (1.62)	
Region of residence			<0.001		<0.001		0.002		0.659		<0.001		<0.001
Rural	402 (22.12)	5.52 (1.55)		5.86 (2.16)		5.49 (2.38)		5.13 (2.43)		6.10 (2.73)		5.38 (1.53)	
Urban	1,415 (77.88)	6.00 (1.73)		6.37 (2.46)		5.94 (2.52)		5.20 (2.52)		6.96 (2.59)		5.95 (1.60)	
Living arrangement			0.044		0.045		0.177		0.051		0.065		0.586
Living alone	233 (12.82)	5.66 (1.94)		5.93 (2.67)		5.64 (2.69)		4.88 (2.56)		6.44 (2.94)		5.77 (1.80)	
Living with others	1,584 (87.18)	5.93 (1.66)		6.31 (2.36)		5.87 (2.47)		5.23 (2.49)		6.82 (2.60)		5.83 (1.58)	
Financial difficulties			0.001		0.012		<0.001		<0.001		0.028		0.714
No	1,402 (77.16)	5.97 (1.70)		6.33 (2.40)		5.96 (2.48)		5.29 (2.55)		6.85 (2.66)		5.83 (1.61)	
Yes	415 (22.84)	5.65 (1.67)		6.00 (2.41)		5.44 (2.53)		4.81 (2.30)		6.52 (2.58)		5.80 (1.59)	
Diabetes duration^a^, year	10.05 (9.19)	5.89 (1.70)	<0.001	6.26 (2.41)	<0.001	5.84 (2.50)	<0.001	5.18 (2.50)	<0.001	6.77 (2.65)	<0.001	5.82 (1.61)	<0.001
Diabetes family history			0.356		0.776		0.164		0.330		0.009		0.216
No	999 (54.98)	5.86 (1.65)		6.24 (2.30)		5.77 (2.45)		5.23 (2.49)		6.63 (2.68)		5.78 (1.60)	
Yes	818 (45.02)	5.93 (1.76)		6.28 (2.53)		5.93 (2.55)		5.12 (2.51)		6.95 (2.59)		5.88 (1.61)	
Diabetes complication			0.001		0.001		0.943		0.005		<0.001		0.028
No	417 (22.95)	5.65 (1.78)		5.92 (2.46)		5.84 (2.56)		4.85 (2.74)		6.32 (2.78)		5.67 (1.65)	
Yes	1,400 (77.05)	5.97 (1.67)		6.36 (2.38)		5.85 (2.48)		5.28 (2.42)		6.91 (2.59)		5.87 (1.59)	
Hypertension			0.002		0.004		0.346		0.008		<0.001		0.080
No	752 (41.39)	5.74 (1.80)		6.06 (2.45)		5.78 (2.58)		4.99 (2.68)		6.49 (2.71)		5.75 (1.64)	
Yes	1,065 (58.61)	6.00 (1.62)		6.39 (2.36)		5.89 (2.44)		5.32 (2.36)		6.97 (2.59)		5.88 (1.58)	
Dyslipidemia			0.002		0.073		0.174		0.057		<0.001		0.002
No	783 (43.09)	5.75 (1.67)		6.14 (2.25)		5.75 (2.54)		5.05 (2.58)		6.47 (2.69)		5.69 (1.62)	
Yes	1,034 (56.91)	6.00 (1.72)		6.34 (2.52)		5.91 (2.46)		5.28 (2.44)		7.00 (2.59)		5.92 (1.59)	
Overweight/obesity			0.001		0.002		0.011		<0.001		0.639		0.655
No	926 (50.96)	6.02 (1.68)		6.43 (2.29)		5.99 (2.43)		5.39 (2.50)		6.80 (2.69)		5.84 (1.62)	
Yes	891 (49.04)	5.76 (1.71)		6.08 (2.51)		5.69 (2.56)		4.97 (2.48)		6.74 (2.60)		5.81 (1.59)	
Antidiabetic medication			<0.001		<0.001		<0.001		<0.001		<0.001		<0.001
None	319 (17.56)	4.77 (1.68)		4.92 (2.54)		5.14 (2.63)		4.28 (2.49)		3.73 (1.86)		5.47 (1.67)	
Oral medication only	685 (37.70)	6.00 (1.65)		6.40 (2.29)		5.92 (2.51)		5.12 (2.45)		7.23 (2.35)		5.84 (1.57)	
Involve injectable medication	813 (44.74)	6.25 (1.56)		6.66 (2.26)		6.05 (2.39)		5.58 (2.45)		7.59 (2.27)		5.95 (1.59)	

^a^ Presented as mean (SD). *SD*, standard deviation.

### Diabetes Self-Management Behaviors and Glycemic Control

The mean score of overall self-management behaviors was 5.89 (SD = 1.70) out of 10, suggesting that participants’ diabetes self-management behaviors were suboptimal. The proportion of suboptimal self-management behaviors in the participants was observed at 50.19%. Among five specific self-management activities, the highest score was found for medication adherence (mean = 6.77, SD = 2.65), followed by dietary control (mean = 6.26, SD = 2.41), physical activity (mean = 5.84, SD = 2.50), physician contact (mean = 5.82, SD = 1.61), and glucose monitoring (mean = 5.18, SD = 2.50) (Table 1). Additionally, we observed that 53.22% of the participants had physical activity of <150 min/week, and most participants chose walking (51.07%) as daily exercise, followed by doing housework (16.02%), fast walking (14.75%), and jogging (10.95%). The mean score of glycemic control was 1.93 (SD = 0.77) out of 3, with 26.86% reporting good glycemic control, suggesting that participants’ glycemic control was suboptimal.

### Impacts of Diabetes Self-Management Behaviors on Glycemic Control and the Heterogeneity

Overall self-management behaviors had a significantly positive impact on glycemic control (M1: coefficient = 0.146, 95% confidence interval [CI] [0.124, 0.167], p < 0.001) after controlling for participant characteristics. Across five specific self-management activities, dietary control (M2: coefficient = 0.099, 95%CI [0.084, 0.114], p < 0.001) showed the greatest positive impact on glycemic control, followed by glucose monitoring (M4: coefficient = 0.092, 95%CI [0.078, 0.106], p < 0.001), physician contact (M6: coefficient = 0.055, 95%CI [0.033, 0.078], p < 0.001), physical activity (M3: coefficient = 0.039, 95%CI [0.025, 0.054], p < 0.001), and medication adherence (M5: coefficient = 0.031, 95%CI [0.014, 0.047], p < 0.001) (Table 2).

**TABLE 2:** The impacts of diabetes self-management behaviors on glycemic control (China. 2023).

Variables	Glycemic control											
	M1		M2		M3		M4		M5		M6	
	Coef [95%CI]	p	Coef [95%CI]	p	Coef [95%CI]	p	Coef [95%CI]	p	Coef [95%CI]	p	Coef [95%CI]	p
Overall self-management behaviors	0.146 [0.124, 0.167]	<0.001	—	—	—	—	—	—	—	—	—	—
Dietary control	—	—	0.099 [0.084, 0.114]	<0.001	—	—	—	—	—	—	—	—
Physical activity	—	—	—	—	0.039 [0.025, 0.054]	<0.001	—	—	—	—	—	—
Glucose monitoring	—	—	—	—	—	—	0.092 [0.078, 0.106]	<0.001	—	—	—	—
Medication adherence	—	—	—	—	—	—	—	—	0.031 [0.014, 0.047]	<0.001	—	—
Physician contact	—	—	—	—	—	—	—	—	—	—	0.055 [0.033, 0.078]	<0.001
Age, year	0 [−0.004, 0.004]	0.945	0 [−0.003, 0.004]	0.790	0.001 [−0.003, 0.005]	0.527	0.002 [−0.002, 0.006]	0.250	0.002 [−0.002, 0.005]	0.431	0.002 [−0.002, 0.006]	0.336
Gender (ref: male)	0.019 [−0.053, 0.090]	0.613	0.01 [−0.061, 0.082]	0.775	0.054 [−0.021, 0.128]	0.157	0.031 [−0.041, 0.103]	0.397	0.049 [−0.026, 0.124]	0.197	0.050 [−0.024, 0.125]	0.185
Marital status (ref: no partner)	−0.061 [−0.196, 0.075]	0.381	−0.068 [−0.204, 0.067]	0.322	−0.073 [−0.213, 0.068]	0.311	−0.033 [−0.169, 0.103]	0.635	−0.061 [−0.203, 0.080]	0.394	−0.063 [−0.204, 0.078]	0.380
Education level (ref: illiterate)												
Primary school	0.079 [−0.116, 0.275]	0.426	0.103 [−0.093, 0.298]	0.304	0.124 [−0.079, 0.327]	0.230	0.07 [−0.126, 0.266]	0.485	0.119 [−0.085, 0.323]	0.253	0.140 [−0.064, 0.343]	0.178
Middle school	0.152 [−0.032, 0.335]	0.105	0.189 [0.006, 0.372]	0.043	0.232 [0.042, 0.422]	0.017	0.145 [−0.039, 0.328]	0.121	0.224 [0.033, 0.415]	0.022	0.261 [0.072, 0.451]	0.007
High school	0.139 [−0.046, 0.324]	0.142	0.18 [−0.005, 0.365]	0.056	0.242 [0.050, 0.434]	0.013	0.143 [−0.043, 0.328]	0.132	0.240 [0.047, 0.433]	0.015	0.270 [0.079, 0.462]	0.006
University or above	0.155 [−0.039, 0.348]	0.117	0.205 [0.012, 0.398]	0.037	0.256 [0.056, 0.456]	0.012	0.14 [−0.054, 0.334]	0.157	0.265 [0.063, 0.466]	0.010	0.303 [0.103, 0.503]	0.003
Medical insurance (ref: no)	−0.144 [−0.358, 0.070]	0.186	−0.123 [−0.337, 0.091]	0.258	−0.146 [−0.368, 0.075]	0.196	−0.143 [−0.357, 0.071]	0.191	−0.157 [−0.379, 0.066]	0.168	−0.142 [−0.364, 0.080]	0.211
Employment status (ref: retired)												
Unemployed	−0.031 [−0.174, 0.112]	0.673	−0.045 [−0.188, 0.097]	0.533	−0.074 [−0.223, 0.074]	0.327	−0.081 [−0.223, 0.062]	0.267	−0.102 [−0.250, 0.047]	0.179	−0.093 [−0.241, 0.056]	0.220
Employed/students	0.025 [−0.080, 0.131]	0.639	0.02 [−0.086, 0.125]	0.715	−0.009 [−0.118, 0.101]	0.875	0.011 [−0.094, 0.117]	0.833	−0.017 [−0.127, 0.092]	0.757	−0.014 [−0.123, 0.096]	0.809
Region of residence (ref: rural)	−0.128 [−0.219, −0.036]	0.006	−0.126 [−0.217, −0.034]	0.007	−0.12 [−0.215, −0.024]	0.014	−0.091 [−0.183, 0.001]	0.053	−0.136 [−0.232, −0.04]	0.005	−0.150 [−0.245, −0.054]	0.002
Living arrangement (ref: living alone)	−0.021 [−0.164, 0.121]	0.769	−0.022 [−0.164, 0.121]	0.766	−0.011 [−0.159, 0.137]	0.884	−0.071 [−0.213, 0.072]	0.331	−0.015 [−0.164, 0.133]	0.842	−0.017 [−0.165, 0.131]	0.824
Financial difficulties (ref: no)	−0.002 [−0.084, 0.081]	0.968	−0.007 [−0.089, 0.076]	0.870	−0.017 [−0.102, 0.069]	0.701	0.008 [−0.075, 0.091]	0.849	−0.023 [−0.109, 0.063]	0.597	−0.033 [−0.118, 0.053]	0.454
Diabetes duration, year	−0.009 [−0.014, −0.005]	<0.001	−0.01 [−0.014, −0.005]	<0.001	−0.008 [−0.013, −0.004]	0.001	−0.008 [−0.013, −0.004]	<0.001	−0.009 [−0.013, −0.004]	<0.001	−0.008 [−0.013, −0.004]	0.001
Diabetes family history (ref: no)	−0.156 [−0.226, −0.086]	<0.001	−0.152 [−0.222, −0.082]	<0.001	−0.168 [−0.240, −0.095]	<0.001	−0.143 [−0.213, −0.073]	<0.001	−0.167 [−0.240, −0.094]	<0.001	−0.165 [−0.237, −0.092]	<0.001
Diabetes complication (ref: no)	−0.034 [−0.117, 0.050]	0.427	−0.042 [−0.125, 0.041]	0.324	−0.027 [−0.113, 0.060]	0.543	−0.052 [−0.136, 0.031]	0.218	−0.034 [−0.121, 0.053]	0.445	−0.040 [−0.127, 0.046]	0.362
Hypertension (ref: no)	0.097 [0.024, 0.170]	0.009	0.094 [0.021, 0.167]	0.012	0.098 [0.022, 0.174]	0.012	0.073 [0, 0.146]	0.050	0.096 [0.020, 0.173]	0.013	0.095 [0.019, 0.171]	0.014
Dyslipidemia (ref: no)	−0.120 [−0.192, −0.049]	0.001	−0.11 [−0.181, −0.038]	0.003	−0.113 [−0.187, −0.039]	0.003	−0.118 [−0.189, −0.047]	0.001	−0.12 [−0.194, −0.045]	0.002	−0.120 [−0.194, −0.046]	0.001
Overweight/obese (ref: no)	−0.029 [−0.098, 0.040]	0.411	−0.032 [−0.101, 0.037]	0.361	−0.046 [−0.118, 0.026]	0.208	−0.028 [−0.097, 0.042]	0.434	−0.057 [−0.129, 0.015]	0.120	−0.053 [−0.125, 0.019]	0.146
Antidiabetic medication (ref: none)												
Oral medication only	0.003 [−0.100, 0.107]	0.950	0.035 [−0.067, 0.138]	0.500	0.13 [0.025, 0.236]	0.015	0.079 [−0.023, 0.181]	0.127	0.053 [−0.065, 0.171]	0.377	0.140 [0.034, 0.245]	0.009
Involve injectable medication	−0.069 [−0.175, 0.037]	0.202	−0.028 [−0.133, 0.077]	0.604	0.077 [−0.030, 0.185]	0.157	0 [−0.105, 0.104]	0.994	−0.003 [−0.124, 0.118]	0.959	0.088 [−0.019, 0.195]	0.107
cons	1.489 [1.125, 1.854]	<0.001	1.621 [1.259, 1.982]	<0.001	1.837 [1.463, 2.212]	<0.001	1.693 [1.333, 2.052]	<0.001	1.952 [1.580, 2.324]	<0.001	1.715 [1.328, 2.102]	<0.001

*Coef* coefficient. *CI*, confidence interval. *ref*, reference group.

The heterogeneous impacts of diabetes self-management behaviors on glycemic control across key groups were explored. In the subgroups of age, overall self-management behaviors and the five specific activities all exerted significantly positive impacts on glycemic control for older participants (coefficient = 0.039–0.155, all p < 0.001) and younger participants (coefficient = 0.023–0.135, all p < 0.05), while the overall effect size was smaller in younger ones. In the subgroups of region of residence, overall self-management behaviors and the five specific activities all significantly promoted glycemic control for urban participants (coefficient = 0.031–0.150, all p < 0.01), while only overall self-management behaviors and dietary control, glucose monitoring, and medication adherence significantly promoted glycemic control for rural participants (coefficient = 0.038–0.135, all p < 0.05). In the subgroups of financial condition, overall self-management behaviors and the five specific activities all significantly improved glycemic control for participants without financial difficulties (coefficient = 0.029–0.147, all p < 0.01), while only overall self-management behaviors and dietary control, physical activity, and glucose monitoring significantly improved glycemic control for participants with financial difficulties (coefficient = 0.038–0.134, all p < 0.05). In general, younger persons, rural persons, and persons with financial difficulties benefited less from diabetes self-management behaviors (Table 3).

**TABLE 3:** Heterogeneous impacts of diabetes self−management behaviors on glycemic control (China. 2023).

	Glycemic control			
	Subgroup 1		Subgroup 2	
	Coef [95%CI]	p	Coef [95%CI]	p
Panel 1: by age	Older participants (≥60 years)		Younger participants (<60 years)	
Overall self−management behaviors	0.155 [0.124, 0.186]	<0.001	0.135 [0.104, 0.166]	<0.001
Dietary control	0.106 [0.085, 0.126]	<0.001	0.092 [0.070, 0.114]	<0.001
Physical activity	0.039 [0.018, 0.059]	<0.001	0.043 [0.022, 0.064]	<0.001
Glucose monitoring	0.075 [0.056, 0.095]	<0.001	0.102 [0.082, 0.123]	<0.001
Medication adherence	0.043 [0.019, 0.067]	<0.001	0.023 [0.001, 0.046]	0.042
Physician contact	0.056 [0.024, 0.087]	<0.001	0.051 [0.019, 0.083]	0.002
Panel 2: by region of residence	Rural participants		Urban participants	
Overall self−management behaviors	0.135 [0.082, 0.188]	<0.001	0.150 [0.126, 0.174]	<0.001
Dietary control	0.097 [0.059, 0.135]	<0.001	0.100 [0.083, 0.116]	<0.001
Physical activity	0.003 [−0.031, 0.037]	0.861	0.050 [0.034, 0.066]	<0.001
Glucose monitoring	0.109 [0.078, 0.140]	<0.001	0.086 [0.070, 0.102]	<0.001
Medication adherence	0.038 [0.003, 0.073]	0.031	0.031 [0.012, 0.049]	0.001
Physician contact	0.026 [−0.026, 0.077]	0.330	0.065 [0.040, 0.090]	<0.001
Panel 3: by financial condition	Participants without financial difficulties		Participants with financial difficulties	
Overall self−management behaviors	0.147 [0.122, 0.172]	<0.001	0.134 [0.086, 0.182]	<0.001
Dietary control	0.100 [0.082, 0.117]	<0.001	0.092 [0.060, 0.125]	<0.001
Physical activity	0.039 [0.022, 0.055]	<0.001	0.038 [0.007, 0.069]	0.017
Glucose monitoring	0.091 [0.075, 0.106]	<0.001	0.090 [0.057, 0.122]	<0.001
Medication adherence	0.029 [0.011, 0.048]	0.002	0.033 [−0.001, 0.067]	0.057
Physician contact	0.057 [0.031, 0.082]	<0.001	0.048 [−0.001, 0.097]	0.054

*Coef* coefficient. *CI*, confidence interval.

### Robustness Tests

To test the results’ robustness, the method of removing one control variable (i.e., diabetes duration) was used and confirmed the positive impacts of overall self-management behaviors and the five specific activities on glycemic control by a coefficient of 0.029–0.144 (all p < 0.01) and the rank of effect size of the activities. Additional test by converting overall self-management behaviors to a categorical variable also verified the results’ robustness (Supplementary Appendix Tables S1, S2).

## Discussion

China has been promoting the national DPCA since July 2019, to improve diabetes management and health of persons with diabetes. However, the COVID-19 outbreak in January 2020 and its induced lockdown and health issues disrupted individuals’ diabetes self-management behaviors and glycemic control. This study was the first, conducted after China dropped COVID-19 lockdown in January 2023, to investigate type 2 diabetes self-management behaviors and glycemic control under the combined impacts of the COVID-19 legacy and the national DPCA, and explore the heterogeneous impacts of five specific self-management activities on glycemic control and how the impacts differ across key groups, thus developing diabetes health promotion interventions.

This study found that diabetes self-management behaviors of our participants was suboptimal with a DSMQ sum score of 5.89, which however was higher than the estimates of 4.85 in China and 4.62–5.25 in the UK during the COVID-19 lockdown [16, 17, 32]. There were 50.19% of our participants reporting suboptimal diabetes self-management behaviors, which was lower than the estimates of 62% in China and 82% in the UK during the COVID-19 lockdown [16, 32], as well as the estimates of 54%–54.8% in China before the COVID-19 pandemic and the national DPCA [6, 7]. These somewhat suggested that although COVID-19 induced lockdown and health issues impeded diabetes self-management behaviors of persons with type 2 diabetes, the effects of the national DPCA were becoming apparent since China dropped COVID-19 lockdown, leading to improvement in diabetes self-management behaviors.

Among five specific self-management activities, medication adherence was found to be the best while glucose-monitoring adherence was the worst. The reasons for higher medication adherence may relate to individuals’ habitual belief of taking medications when sick; the directly perceivable effects of medications due to their quick and direct impacts on controlling diseases; the affordability of medications as most are covered by medical insurance; and the ease of medication management as technological products (e.g., phone apps, smart pillboxes) can remind individuals to take medication without largely modifying their lifestyles [5, 8, 33, 34]. The reasons for lower glucose-monitoring adherence may relate to individuals’ low awareness of the importance of glucose monitoring; habitual belief of taking medications is enough; fears of pain, blood, or needles; and frugal lifestyle habits or financial barriers as glucose monitoring equipment is not covered by medical insurance [5, 6, 35, 36]. Employed persons may not have enough time to test glucose regularly, or feel embarrassed or inconvenient to test glucose in working environments [8, 35]. These suggested that health promotion interventions on glucose monitoring should be strengthened, financial supports should be provided for persons in need, and attentions should be paid to improving the working environments, such as creating private spaces. Besides, we observed that 53.22% of our participants had physical activity <150 min/week and 51.07% chose walking as daily exercise. This did not meet the requirement of Chinese guideline which recommends persons with type 2 diabetes to engage in ≥150 min/week of moderate intensity aerobic exercise [37]. These suggested that health promotion interventions should not only encourage individuals to exercise regularly, but also inspire them to guarantee sufficient exercise duration and intensity. Overall, our findings illustrated that differences existed in individuals’ adherence to diabetes self-management activities. Thus, customized diabetes health education interventions should be inspired, focusing on addressing the deficiencies or weaknesses in individuals’ specific self-management activities after assessment, rather than blindly providing tedious or complex interventions covering all self-management activities.

About 26.86% of our participants reported having good glycemic control. This proportion was higher than the estimate of 16.7% during the COVID-19 lockdown [16], as well as the estimate of 14.7%–22.97% before the COVID-19 pandemic and the national DPCA in China [9–11]. This somewhat suggested that glycemic control in Chinese persons with type 2 diabetes was suboptimal, but may be gradually improving under the combined impacts of the COVID-19 legacy and the national DPCA. Although COVID-19-induced suboptimal self-management behaviors and health issues negatively impacted individuals’ glycemic control, the effects of the national DPCA have been emerging since China dropped COVID-19 lockdown.

This study demonstrated that overall self-management behaviors and the five specific activities all had positive impacts on improving glycemic control, with dietary control showing the greatest impact but medication adherence the least. On one hand, this provided empirical support for the clinical guidelines that advocate persistent diabetes self-management behaviors, further reinforcing the basis for self-management as a core component of diabetes management. Using empirical data, this study also offered a detailed evaluation of the impacts of five key self-management activities on glycemic control. Despite the variations in the impacts across five activities, the results provided healthcare providers with valuable data needed to guide individualized self-management plans, enabling more precise, person-centered, and evidence-based diabetes management. Moreover, the quantitative data can help individuals visually understand the importance of diabetes self-management behaviors, thus motivating them to adhere to self-management. On the other hand, the results recommended that individuals’ glycemic control not only depends on medications but also, importantly, necessitates individuals cultivating and maintaining healthy lifestyles, such as a healthy diet. Therefore, healthcare providers should pay more attention to help individuals foster and maintain healthy lifestyles by offering practical, tailored advice and ongoing support, including individualized nutritional and exercise guidance, empowering individuals through education, encouraging gradual changes, addressing barriers, involving family support, and monitoring progress.

As healthcare increasingly prioritizes population health outcomes and value-based care, social determinants of health (SDOH) have become essential intervention targets for achieving health equity [38]. In this context, identifying key groups that require more support in diabetes self-management based on SDOH in type 2 diabetes is crucial. Targeted health promotion interventions for these groups can enhance intervention efficiency, optimize resource allocation, and ultimately improve self-management behaviors at the entire population level by enhancing self-management behaviors of these groups. Thus, we explored how the impacts of diabetes self-management behaviors on glycemic control differ across several subgroups of participants that represent key SDOH in type 2 diabetes. Given the rising trend of diabetes in younger population, we compared the impacts of diabetes self-management behaviors on glycemic control between younger and older participants. It was found that diabetes self-management behaviors conferred smaller impacts on improving glycemic control of younger participants. Consistent with previous studies, our younger participants reported poorer diabetes self-management behaviors than older ones, which may be a reason for the difference [7, 27, 28]. Older persons often have a longer diabetes duration and poorer body function, thus facing a greater risk of complications, which makes them place more emphasis on diabetes self-management; additionally, most of them have retired, thus having more time to manage their diabetes [39, 40]. Conversely, younger persons are often busier with work and socializing, and face more temptations; and most of them have a shorter diabetes duration and thus have a lower risk of complications [8]. Consequently, they may have less time, willpower, and awareness for managing their diabetes [41]. Besides, because older persons typically have higher glucose levels than younger ones, the same diabetes self-management behaviors may have a greater impact on glycemic control in older persons. Considering the uneven developments between rural and urban China, this study investigated the rural-urban difference in the impacts of diabetes self-management behaviors on glycemic control. It was found that rural participants benefited less from diabetes self-management behaviors. This may be somewhat because our rural participants had poorer diabetes self-management behaviors than urban ones. Compared with urban persons, rural persons may have lower education levels, more economic constraints, and less access to medical resources. As a result, they may have lower awareness and capability for diabetes self-management, and thus have lower adherence to self-management behaviors. Poor economic status was found to link to poor diabetes self-management [27, 42]. This study found that participants with financial difficulties had poorer diabetes self-management behaviors and benefited less from self-management behaviors in terms of glycemic control, compared with those without financial difficulties. Persons with financial difficulties may spend more time struggling to make ends meet and improve their living standards, thus lacking sufficient time, energy, and awareness for diabetes self-management. Besides, although most Chinese persons have basic medical insurance [43], those with financial difficulties are more likely to be uninsured. Moreover, medical insurance does not cover the cost of glucose monitoring equipment, which may increase individuals’ economic burden. Consequently, persons with financial difficulties may struggle to afford their medications and glucose equipment.

Continuous efforts by multiple stakeholders are needed to support the national DPCA and engage in health promotions for improving diabetes self-management and glycemic control of persons with type 2 diabetes. The government should provide policy, resource, and financial supports to encourage communities, healthcare institutions, employers, and families to engage in diabetes health promotion activities and to motivate individuals to adhere to diabetes self-management. The communities and healthcare institutions should intensify health educations, interventions, and supports for persons with type 2 diabetes, especially younger ones, rural ones, and those with financial difficulties, to enhance their knowledge, awareness, and capacity of diabetes self-management. The employers should provide employees with healthy diet and private spaces, and implement rewarded campaigns to nudge employees to exercise regularly. The families should provide more emotional and instrument supports for persons with type 2 diabetes. Meanwhile, health promotion interventions in different population groups should be customized, taking account of individualized obstacles and characteristics.

This study has several limitations. First, we used a convenient sampling strategy, which may limit the sample’s representativeness and result in selection bias. Fortunately, we included a nationwide sample which may reduce the bias. Second, this was a cross-sectional survey that was difficult to clarify the causal relationships between self-management behaviors and glycemic control. Third, the use of a self-reported design may introduce biases such as recall bias, social desirability bias, and variability in interpretation. For example, glycemic control is self-reported by participants, which may be influenced by recall bias or subject to varied interpretation by participants. Fourth, this survey started 3 months after the lockdown ended and a carryover effect of the lockdown may have been reflected in self-management behaviors and glycemic control. This may limit the representativeness of the findings regarding the post-COVID status of type 2 diabetes. However, since we targeted the combined impacts of the COVID-19 legacy (including lockdown legacy) and the DPCA and the survey spanned several months, this may have partially mitigated the potential bias. Fifth, income, as an important indicator of socioeconomic status, was not included as a covariate, which may have introduced confounding bias. However, we controlled for financial difficulties as a proxy, which may have partially mitigated the bias.

In conclusion, this study found that diabetes self-management behaviors and glycemic control of persons with type 2 diabetes in China were suboptimal. However, when compared to evidence from before and during the COVID-19 pandemic and the implementation of the DPCA, the findings suggested a potential improvement in both outcomes. This somewhat presented the phased achievements of the national DPCA, which mitigated the harms of COVID-19. Among five specific self-management activities, medication adherence was the best but glucose-monitoring adherence was the worst. This suggested that differences existed in individuals’ adherence to self-management activities, thus, customized health promotions should focus on addressing the deficiencies in individuals’ self-management activities after assessment rather than blindly covering all activities. Overall self-management behaviors and the five specific activities all improved glycemic control, with dietary control showing the greatest impact while medication adherence the least. This provided empirical evidence for clinical guidelines and recommended that glycemic control not only depends on medications but also, importantly, necessitates individuals maintaining healthy lifestyles, such as a healthy diet. Younger persons, rural persons, and persons with financial difficulties had lower adherence to and benefited less from diabetes self-management behaviors, suggesting that they are key groups that need more support. Our study holds valuable implications, as it not only checked the outcomes of the national DPCA, but also provided healthcare providers with empirical evidence for guiding individualized self-management plans and informing the key groups that need more support, thus allocating resources more efficiently. Our suggestions can be extended to other chronic diseases.
