# Supplementary material for: Type 2 Diabetes Self-Management Behaviors and Glycemic Control Under China’s Diabetes Prevention and Control Action Program
Source: Int J Public Health. 2025 May 30;70:1608067. doi: 10.3389/ijph.2025.1608067 (PMC12162350; doi:10.3389/ijph.2025.1608067)
Supplement: Supplementary file 1 [file DataSheet1.docx]

Table S1. The impacts of diabetes self-management behaviors on glycemic control by removing the control variable of diabetes duration

|  | Glycemic control | | | | | | | | | | | |
| --- | --- | --- | --- | --- | --- | --- | --- | --- | --- | --- | --- | --- |
|  | M1 | | M2 | | M3 | | M4 | | M5 | | M6 | |
| Variables | Coef [95%CI] | p | Coef [95%CI] | p | Coef [95%CI] | p | Coef [95%CI] | p | Coef [95%CI] | p | Coef [95%CI] | p |
| Overall self-management behaviors | 0.144 [0.122, 0.166] | <0.001 |  |  |  |  |  |  |  |  |  |  |
| Dietary control |  |  | 0.097 [0.082, 0.113] | <0.001 |  |  |  |  |  |  |  |  |
| Physical activity |  |  |  |  | 0.039 [0.024, 0.054] | <0.001 |  |  |  |  |  |  |
| Glucose monitoring |  |  |  |  |  |  | 0.091 [0.077, 0.105] | <0.001 |  |  |  |  |
| Medication adherence |  |  |  |  |  |  |  |  | 0.029 [0.013, 0.045] | 0.001 |  |  |
| Physician contact |  |  |  |  |  |  |  |  |  |  | 0.054 [0.032, 0.076] | <0.001 |
| Age, year | -0.002 [-0.006, 0.001] | 0.176 | -0.002 [-0.005, 0.002] | 0.297 | -0.001 [-0.004, 0.003] | 0.654 | 0 [-0.003, 0.004] | 0.968 | -0.001 [-0.004, 0.003] | 0.765 | 0 [-0.004, 0.003] | 0.905 |
| Gender (ref: male) | 0.026 [-0.046, 0.098] | 0.482 | 0.018 [-0.054, 0.09] | 0.620 | 0.06 [-0.015, 0.134] | 0.115 | 0.037 [-0.035, 0.109] | 0.311 | 0.056 [-0.019, 0.131] | 0.144 | 0.057 [-0.018, 0.131] | 0.137 |
| Marital status (ref: no partner) | -0.063 [-0.199, 0.073] | 0.361 | -0.071 [-0.207, 0.065] | 0.305 | -0.075 [-0.216, 0.066] | 0.297 | -0.035 [-0.172, 0.101] | 0.610 | -0.064 [-0.206, 0.078] | 0.376 | -0.066 [-0.207, 0.076] | 0.363 |
| Education level (ref: illiterate) |  |  |  |  |  |  |  |  |  |  |  |  |
| Primary school | 0.091 [-0.106, 0.287] | 0.366 | 0.114 [-0.082, 0.311] | 0.255 | 0.134 [-0.07, 0.337] | 0.198 | 0.079 [-0.117, 0.276] | 0.428 | 0.129 [-0.075, 0.334] | 0.215 | 0.149 [-0.055, 0.353] | 0.151 |
| Middle school | 0.173 [-0.01, 0.357] | 0.064 | 0.211 [0.028, 0.395] | 0.024 | 0.251 [0.061, 0.441] | 0.010 | 0.164 [-0.02, 0.347] | 0.081 | 0.244 [0.053, 0.436] | 0.012 | 0.28 [0.09, 0.47] | 0.004 |
| High school | 0.16 [-0.026, 0.346] | 0.092 | 0.201 [0.016, 0.387] | 0.033 | 0.26 [0.068, 0.452] | 0.008 | 0.16 [-0.026, 0.346] | 0.091 | 0.26 [0.066, 0.453] | 0.008 | 0.288 [0.096, 0.479] | 0.003 |
| University or above | 0.171 [-0.023, 0.365] | 0.084 | 0.221 [0.028, 0.415] | 0.025 | 0.27 [0.07, 0.47] | 0.008 | 0.153 [-0.041, 0.348] | 0.122 | 0.28 [0.078, 0.481] | 0.007 | 0.316 [0.116, 0.517] | 0.002 |
| Medical insurance (ref: no) | -0.15 [-0.365, 0.064] | 0.170 | -0.13 [-0.345, 0.085] | 0.235 | -0.152 [-0.374, 0.071] | 0.181 | -0.148 [-0.363, 0.066] | 0.176 | -0.162 [-0.385, 0.062] | 0.157 | -0.148 [-0.37, 0.075] | 0.194 |
| Employment status (ref: retired) |  |  |  |  |  |  |  |  |  |  |  |  |
| Unemployed | -0.023 [-0.166, 0.12] | 0.753 | -0.037 [-0.181, 0.106] | 0.608 | -0.067 [-0.216, 0.082] | 0.379 | -0.073 [-0.216, 0.07] | 0.317 | -0.094 [-0.243, 0.055] | 0.214 | -0.085 [-0.234, 0.063] | 0.261 |
| Employed/students | 0.035 [-0.07, 0.141] | 0.513 | 0.03 [-0.076, 0.136] | 0.579 | 0.001 [-0.109, 0.11] | 0.992 | 0.021 [-0.085, 0.126] | 0.699 | -0.008 [-0.118, 0.102] | 0.888 | -0.004 [-0.114, 0.105] | 0.941 |
| Region of residence (ref: rural) | -0.14 [-0.232, -0.049] | 0.003 | -0.139 [-0.231, -0.047] | 0.003 | -0.131 [-0.226, -0.036] | 0.007 | -0.103 [-0.195, -0.01] | 0.029 | -0.147 [-0.243, -0.052] | 0.003 | -0.161 [-0.257, -0.065] | 0.001 |
| Living arrangement (ref: living alone) | -0.007 [-0.15, 0.136] | 0.922 | -0.007 [-0.15, 0.136] | 0.922 | 0.001 [-0.147, 0.15] | 0.984 | -0.058 [-0.201, 0.085] | 0.427 | -0.003 [-0.151, 0.146] | 0.973 | -0.004 [-0.152, 0.144] | 0.956 |
| Financial difficulties (ref: no) | -0.003 [-0.086, 0.08] | 0.942 | -0.008 [-0.091, 0.075] | 0.844 | -0.018 [-0.104, 0.068] | 0.684 | 0.007 [-0.076, 0.09] | 0.869 | -0.024 [-0.111, 0.062] | 0.578 | -0.034 [-0.12, 0.052] | 0.443 |
| Diabetes family history (ref: no) | -0.178 [-0.247, -0.108] | <0.001 | -0.175 [-0.244, -0.106] | <0.001 | -0.187 [-0.259, -0.116] | <0.001 | -0.163 [-0.232, -0.093] | <0.001 | -0.187 [-0.259, -0.115] | <0.001 | -0.185 [-0.256, -0.113] | <0.001 |
| Diabetes complication (ref: no) | -0.042 [-0.125, 0.042] | 0.330 | -0.05 [-0.133, 0.034] | 0.243 | -0.034 [-0.121, 0.053] | 0.444 | -0.059 [-0.143, 0.024] | 0.164 | -0.041 [-0.128, 0.046] | 0.353 | -0.047 [-0.134, 0.04] | 0.286 |
| Hypertension (ref: no) | 0.092 [0.019, 0.165] | 0.014 | 0.089 [0.016, 0.162] | 0.017 | 0.093 [0.017, 0.169] | 0.016 | 0.069 [-0.004, 0.142] | 0.065 | 0.092 [0.015, 0.168] | 0.018 | 0.09 [0.014, 0.167] | 0.020 |
| Dyslipidemia (ref: no) | -0.125 [-0.196, -0.053] | 0.001 | -0.114 [-0.186, -0.043] | 0.002 | -0.117 [-0.191, -0.043] | 0.002 | -0.122 [-0.193, -0.05] | 0.001 | -0.123 [-0.198, -0.049] | 0.001 | -0.124 [-0.198, -0.05] | 0.001 |
| Overweight/obese (ref: no) | -0.018 [-0.087, 0.051] | 0.608 | -0.021 [-0.09, 0.048] | 0.552 | -0.036 [-0.108, 0.036] | 0.323 | -0.018 [-0.087, 0.052] | 0.618 | -0.047 [-0.119, 0.025] | 0.204 | -0.043 [-0.115, 0.029] | 0.239 |
| Antidiabetic medication (ref: none) |  |  |  |  |  |  |  |  |  |  |  |  |
| Oral medication only | -0.021 [-0.124, 0.083] | 0.696 | 0.011 [-0.092, 0.113] | 0.838 | 0.107 [0.003, 0.212] | 0.044 | 0.056 [-0.045, 0.158] | 0.276 | 0.035 [-0.083, 0.152] | 0.563 | 0.117 [0.012, 0.221] | 0.029 |
| Involve injectable medication | -0.126 [-0.229, -0.024] | 0.016 | -0.087 [-0.188, 0.014] | 0.093 | 0.024 [-0.079, 0.128] | 0.641 | -0.054 [-0.154, 0.047] | 0.295 | -0.052 [-0.171, 0.066] | 0.387 | 0.035 [-0.068, 0.138] | 0.510 |
| cons | 1.566 [1.203, 1.93] | <0.001 | 1.699 [1.339, 2.06] | <0.001 | 1.903 [1.529, 2.276] | <0.001 | 1.757 [1.398, 2.116] | <0.001 | 2.02 [1.648, 2.391] | <0.001 | 1.785 [1.398, 2.171] | <0.001 |

*Coef* coefficient. *CI* confidence interval. *ref* reference group.

Table S2. The impact of overall self-management behaviors (poor, good) on glycemic control

|  | Glycemic Control | |
| --- | --- | --- |
| Variables | Coef [95%CI] | p |
| Overall self-management behaviors* (ref: poor) | 0.384 [0.31, 0.457] | <0.001 |
| Age, year | 0 [-0.004, 0.004] | 0.946 |
| Gender (ref: male) | 0.032 [-0.041, 0.105] | 0.392 |
| Marital status (ref: no partner) | -0.074 [-0.212, 0.063] | 0.290 |
| Education level (ref: illiterate) |  |  |
| Primary school | 0.098 [-0.101, 0.297] | 0.332 |
| Middle school | 0.178 [-0.008, 0.364] | 0.061 |
| High school | 0.174 [-0.014, 0.363] | 0.070 |
| University or above | 0.192 [-0.004, 0.389] | 0.055 |
| Medical insurance (ref: no) | -0.139 [-0.356, 0.078] | 0.210 |
| Employment status (ref: retired) |  |  |
| Unemployed | -0.064 [-0.209, 0.081] | 0.386 |
| Employed/students | 0.004 [-0.103, 0.111] | 0.937 |
| Region of residence (ref: rural) | -0.134 [-0.227, -0.04] | 0.005 |
| Living arrangement (ref: living alone) | -0.002 [-0.147, 0.143] | 0.978 |
| Financial difficulties (ref: no) | -0.016 [-0.1, 0.068] | 0.714 |
| Diabetes duration, year | -0.009 [-0.013, -0.004] | <0.001 |
| Diabetes family history (ref: no) | -0.164 [-0.235, -0.093] | <0.001 |
| Diabetes complication (ref: no) | -0.034 [-0.119, 0.05] | 0.426 |
| Hypertension (ref: no) | 0.105 [0.031, 0.179] | 0.006 |
| Dyslipidemia (ref: no) | -0.116 [-0.188, -0.044] | 0.002 |
| Overweight/obese (ref: no) | -0.037 [-0.107, 0.033] | 0.300 |
| Antidiabetic medication (ref: none) |  |  |
| Oral medication only | 0.069 [-0.035, 0.173] | 0.194 |
| Involve injectable medication | 0.006 [-0.1, 0.112] | 0.914 |
| _cons | 2.051 [1.688, 2.413] | <0.001 |

*Coef* coefficient. *CI* confidence interval. *ref* reference group.

* Overall self-management behavior is converted to a categorical variable (poor, good) based on the recommended cut-off score of ≤6.0 of the Diabetes Self-Management Questionnaire.
